# Implementation and validation of a competency assessment tool for laparoscopic cholecystectomy

**DOI:** 10.1007/s00464-022-09264-0

**Published:** 2022-06-15

**Authors:** Mickael Chevallay, Emilie Liot, Ian Fournier, Ziad Abbassi, Andrea Peloso, Monika E. Hagen, Stefan P. Mönig, Philippe Morel, Christian Toso, Nicolas Buchs, Danilo Miskovic, Frederic Ris, Minoa K. Jung

**Affiliations:** 1 Division of Digestive Surgery, Department of Surgery, Geneva University Hospital and Faculty of Medicine, Rue Gabrielle-Perret-Gentil 4, 1211 Geneva, Switzerland; 2 Department of Surgery and Cancer, Imperial College, St Mary’s Hospital, London, UK

## Abstract

**Background:**

Achieving proficiency in a surgical procedure is a milestone in the career of a trainee. We introduced a competency assessment tool for laparoscopic cholecystectomy in our residency program. Our aim was to assess the inter-rater reliability of this tool.

**Methods:**

We included all laparoscopic cholecystectomies performed by residents under the supervision of board certified surgeons. All residents were assessed at the end of the procedure by the supervising surgeon (live reviewer) using our competency assessment tool. Video records of the same procedure were analyzed by two independent reviewers (reviewer A and B), who were blinded to the performing trainee’s. The assessment had three parts: a laparoscopic cholecystectomy-specific assessment tool (LCAT), the objective structured assessment of technical skills (OSATS) and a 5-item visual analogue scale (VAS) to address the surgeon’s autonomy in each part of the cholecystectomy. We compared the assessment scores of the live supervising surgeon and the video reviewers.

**Results:**

We included 15 junior residents who performed 42 laparoscopic cholecystectomies. Scoring results from live and video reviewer were comparable except for the OSATS and VAS part. The score for OSATS by the live reviewer and reviewer B were 3.68 vs. 4.26 respectively (*p* = 0.04) and for VAS (5.17 vs. 4.63 respectively (*p* = 0.03). The same difference was found between reviewers A and B with OSATS score (3.75 vs. 4.26 respectively (*p* = 0.001)) and VAS (5.56 vs. 4.63 respectively; *p* = 0.004)).

**Conclusion:**

Our competency assessment tool for the evaluation of surgical skills specific to laparoscopic cholecystectomy has been shown to be objective and comparable in-between raters during live procedure or on video material.

**Supplementary Information:**

The online version contains supplementary material available at 10.1007/s00464-022-09264-0.

Acquisition and improvement of operative skills are central in surgical training. The path from a junior trainee to an independent surgeon involves receiving evaluation and correction from experienced mentors. In most European countries, Australia, and North America, certification in general surgery is based on a minimal required number of procedures without a benchmark of the trainee’s autonomy and competence [1]. The American Board of Surgery (ABS) requires six operative performance assessments during residency that are chosen from 13 interventions. However, there is no requirement to achieve a minimal score. As an exception, the certificate of completion of training (CCT) in general surgery in the United Kingdom requires that three different consultant mentors confirm the trainee’s ability to perform three different procedures without supervision [2].

A worrying number of surgeons might finish their training without sufficient surgical skills to perform basic procedures such as laparoscopic cholecystectomy. Furthermore, the legal restriction of working hours [3] clashes with the need to obtain competence and autonomy in different surgeries at the end of a resident’s training program. An improvement in the efficiency of the educational system in surgery is required. An objective assessment tool for surgical skills could provide such an improvement so that confidence in an intervention does not rely solely on an individual’s perception and self-assessment. We adopted the laparoscopic competency assessment tool (CAT) validated in colorectal surgery [4] and modified it for standard cholecystectomy. The essential steps of a laparoscopic colectomy were replaced by those of a laparoscopic cholecystectomy. This study aimed to evaluate the feasibility of implementation of this assessment tool, its objectivity and inter-rater reliability on different supports.

## Methods

We included all patients with cholecystectomies fully performed by trainees from our hospital’s visceral surgery training program between June 2013 and January 2016. The interventions were performed either in an emergency or an elective setting for uncomplicated symptomatic gallstone disease or acute cholecystitis. Surgery was performed under the supervision of a senior surgeon with board certification. Procedures which were not performed entirely by the trainee or for which the video recording was not available were excluded. If the senior surgeon felt that significant oral guidance was given during the surgery, this was mentioned at the bottom of the assessment sheet and the procedure was excluded from the analysis. We collected the different scores prospectively. Patient’s demographic data (age, sex, body mass index [BMI], American Society of Anesthesiologists [ASA] score) and the level of case complexity as rated by the senior surgeon were reported. The senior surgeon who had the role of the live reviewer completed the assessment tool form immediately after the surgery and gave feedback to the trainee. Each procedure was recorded, and the video material was collected at the end of the surgery. Each video received a randomized number and was anonymized for the patient, the live reviewer, and the trainee. One investigator kept a list of matches between the video number and the live assessment form. Two blinded senior surgeons designated as A and B separately reviewed the video recordings of each surgery and filled out the LCAT (except for the cholangiography item which was not shown in the video). The video reviewers did not participate in the live evaluation.

All procedures performed in this study met the ethical standards of the institutional research committee, and the 1964 Declaration of Helsinki and its later amendments or comparable ethical standards. Institutional Review Board was consulted and informed consent was waived. For this type of study, formal consent was not required.

### Assessment tool

Our assessment tool was composed of three parts.

The first part was the Laparoscopic Competency Assessment Tool for laparoscopic cholecystectomy (LCAT) adapted from the CAT in colorectal surgery (4). The CAT designed for laparoscopic colectomy divides the surgery into four steps: exposure, control of the vessels, mobilisation and resection/anastomosis. In each step reviewers evaluate the use of instruments, manipulation of tissues, complications and end result.

**Fig. 1:**
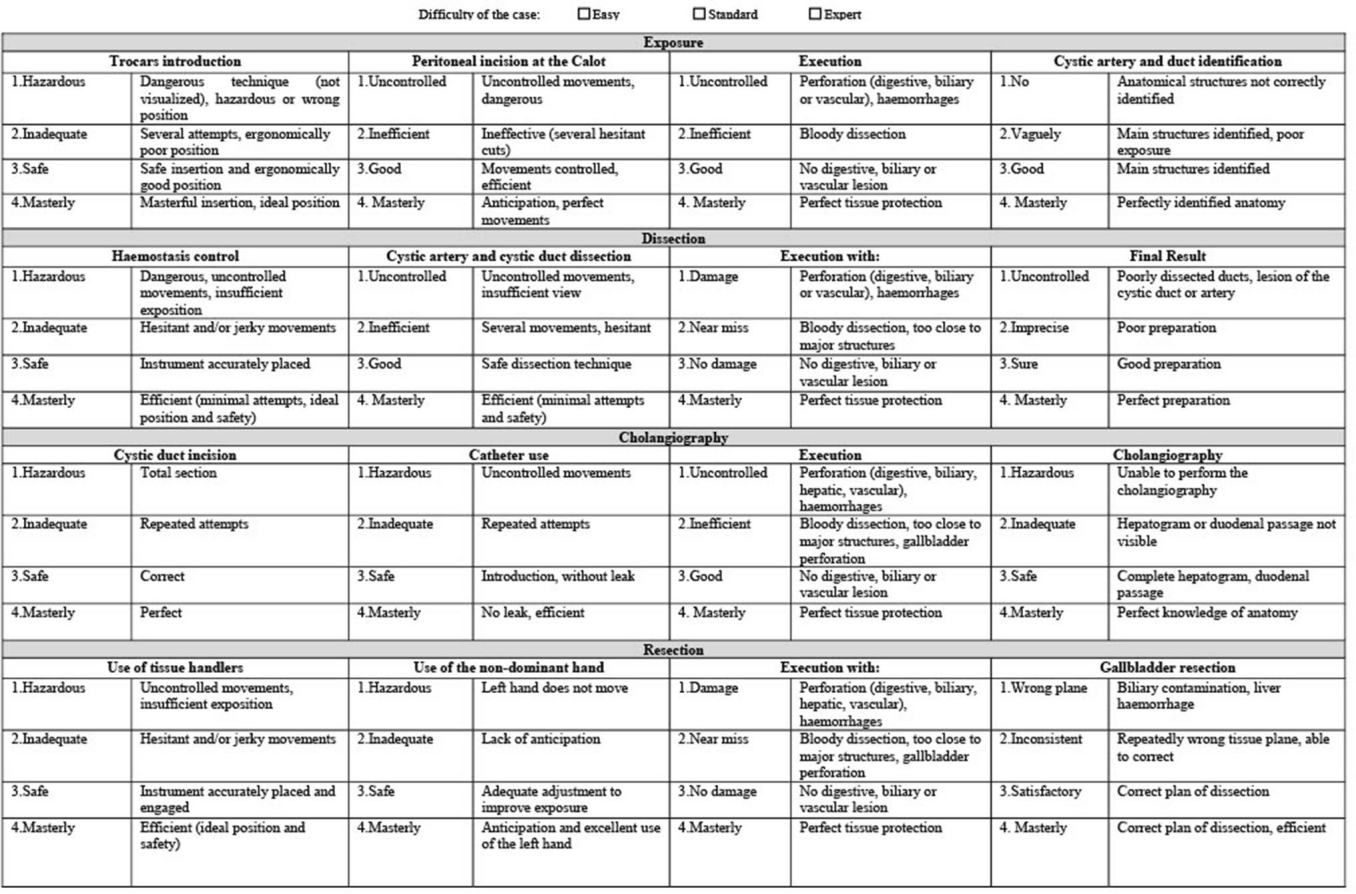
The laparoscopic competency assessment tool for cholecystectomy with the four key steps of the procedure

Two senior surgeons (M.J. and F.R.) identified four major steps of a standard laparoscopic cholecystectomy: (1) exposition of the surgical field, (2) dissection of Calot’s triangle, (3) cholangiography and (4) resection of the gallbladder (Fig. 1). Each of the steps was subdivided into four tasks that received a rating from 1 to 4 (1 = dangerous, 4 = masterly) (Fig. 1). For each step, an overall score was calculated by averaging the ratings received for the four tasks. Each task corresponds to an essential stage of cholecystectomy that needs to be completed before moving to the next one. The sequence of the assessment items follows the course of a laparoscopic cholecystectomy. The adapted CAT was reviewed and validated by eight senior laparoscopic surgeons in our department. A supplementary video demonstrates the assessment of the different steps including examples of different scoring levels (Supplementary Video). One item was not assessed by the video reviewers (cholangiography interpretation) because it was not captured in the recordings.

**Fig. 2:**
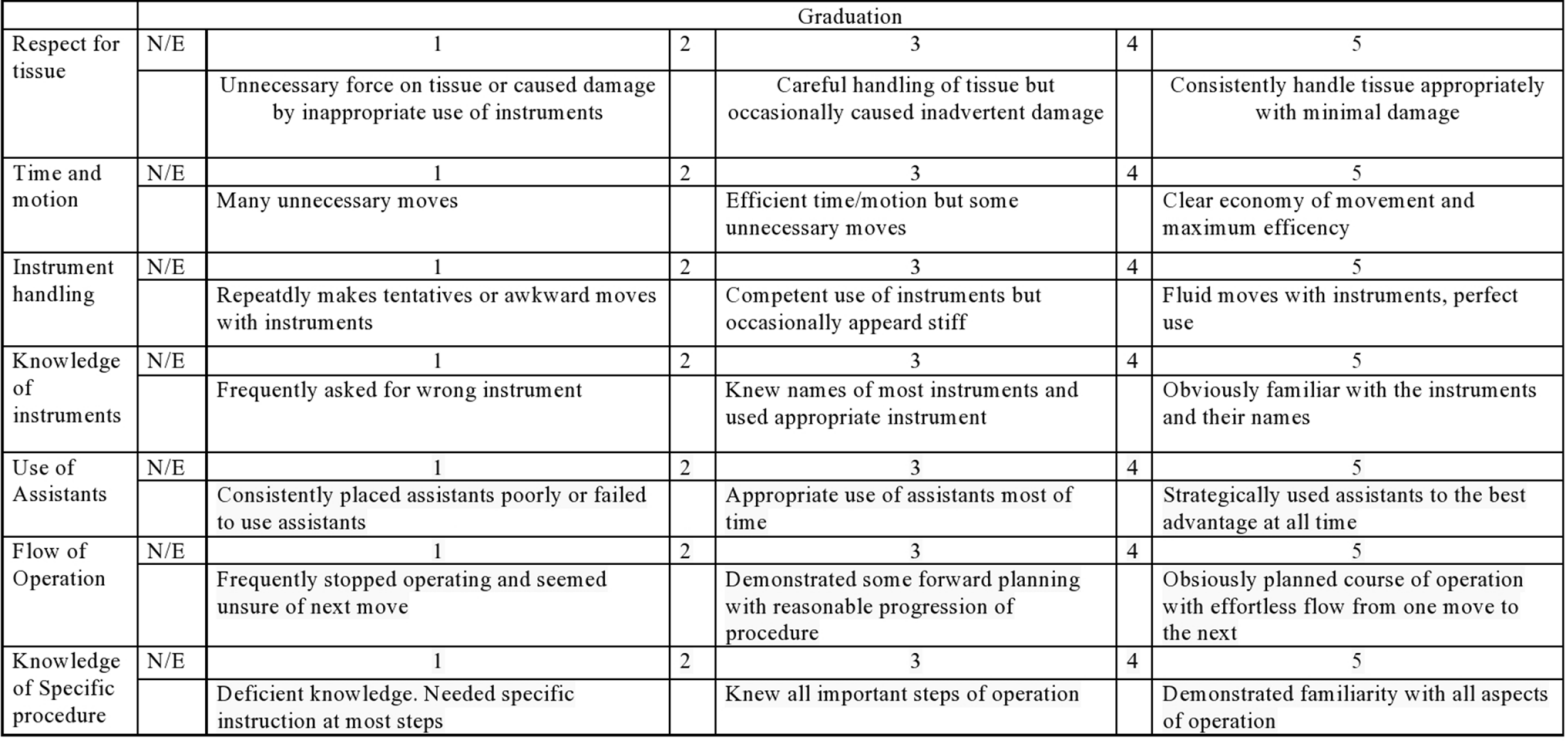
Objective structured assessment of technical skills (OSATS). Graduation from 1 (worst) to 5 (best) for general surgical skills during laparoscopic cholecystectomy

The second part was the validated objective structured assessment of technical skills (OSATS) (13) for general surgical skill assessment (Fig. 2).

**Fig. 3:**
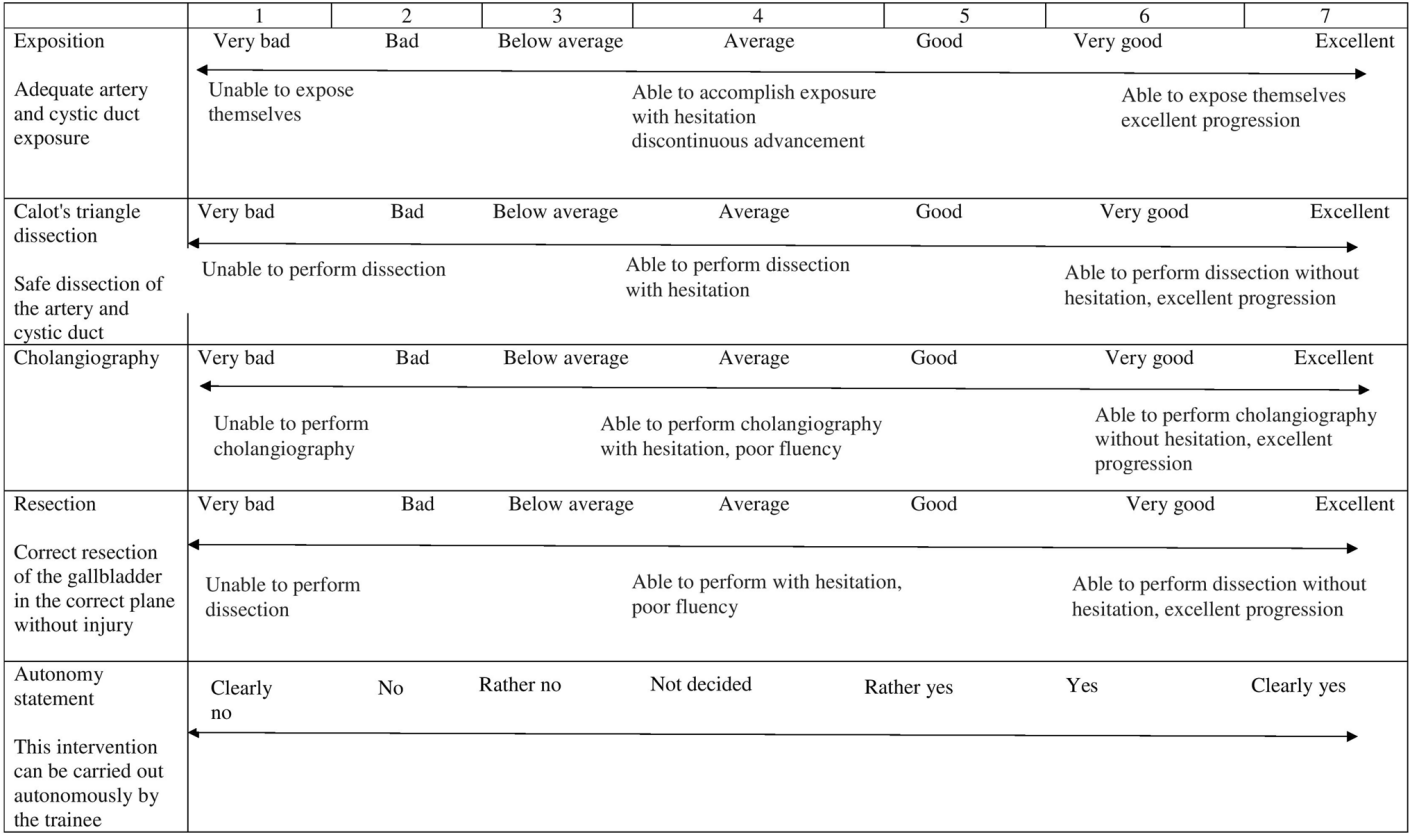
Visual analogue scale (VAS) for laparoscopic cholecystectomy for each step and general autonomy statement, rating from 1 (very bad) to 7 (excellent)

The third part was a visual analogue scale (VAS) assessment of the capacity of the trainee to perform each step (exposition, Calot’s dissection, cholangiography, and resection) without supervision on a scale from 1 (clearly not) to 7 (clearly yes). An overall mean score for the VAS was calculated from the sum of ratings for each step. A global VAS for the autonomy during the laparoscopic cholecystectomy completed the score (Fig. 3). To obtain autonomy for the procedure, a minimum score of 5 on the VAS is required.

### Statistical analysis

We compared the means for each step between reviewers using Student’s *t* test for categorical data. Statistical significance was assumed when *p* value was inferior to 0.05. Confidence intervals (CI) were set at 95%. For live-reviewed assessment, we calculated a Pearson’s correlation coefficient between the operative time and the score for each step. To determine the inter-rater reliability of the assessment tool items, we calculated intraclass correlation coefficients (ICCs) for three ratings: one given during the surgery by the supervisor and two given by the video reviewers. ICC estimates and their 95% confidence intervals were based on a mean-rating (*k* = 3), absolute-agreement, two-way random model, single measure. Values inferior to 0.5, between 0.5 and 0.75, between 0.75 and 0.9, and superior to 0.90 were indicative of poor, moderate, good, and excellent reliability respectively [5]. All analyses were performed using the Statistical Package for the Social Sciences software (version 22.0.0, SPSS Inc, Chicago, IL).

## Results

We recorded 42 laparoscopic cholecystectomies performed by 15 trainees. Eight trainees had from 1 to 3 years of post-graduate experience and seven trainees from 4 to 6 years of post-graduate experience. 30 procedures were performed by ten trainees (3 laparoscopic cholecystectomy per trainee), 8 procedures by four trainees (2 procedures per trainee) and 4 were performed by one trainee.

All interventions were supervised by one of 15 senior surgeons. The demographic data of the 42 patients are shown in Table 1. No conversion to laparotomy was observed.

**Table 1:** Demographics data

Interventions, *N*	42
Age in years, mean (± SD)	52 (± 17)
Female *N* (%)	32 (75%)
Level of complexity	
Easy *N* (%)	12 (32%)
Standard *N* (%)	21 (55%)
Expert, *N* (%)	5 (13%)
ASA	
I *N* (%)	9 (22%)
II *N* (%)	26 (62%)
III *N* (%)	8 (16%)
BMI, mean (± SD)	26.63 (± 5.64)
Operative time in minutes, mean (± SD)	105 (± 35)

*SD* standard deviation, *ASA* American Society of Anesthesiologists score, *BMI* Body Mass Index

### Live and video evaluations

The mean score for each step of the competency assessment tool was reported for the live reviewer and the two video reviewers. The most completed items were the autonomy score and Calot’s dissection (completed in 42/42 procedures for all reviewers). The mean scores for each step are presented in Table 2.

**Table 2:** Mean scores in each step of the assessment tool from the three reviewers

LCAT step	Reviewer live		Reviewer A		Reviewer B	
	*N*	Mean (± SD)	*N*	Mean (± SD)	*N*	Mean (± SD)
Exposure	42	3.14 (± 0.46)	42	3.19 (± 0.46)	41	3.11 (± 0.61)
Calot dissection	42	3.14 (± 0.55)	42	3.07 (± 0.48)	42	2.94 (± 0.67)
Cholangiography	36	3.32 (± 0.53)	39	3.26 (± 0.54)	39	3.23 (± 0.74)
Resection	42	3.1 (± 0.61)	42	3.12 (± 0.63)	39	2.9 (± 0.69)
OSATS	40	3.68 (± 0.88)	42	3.75 (± 0.75)	39	4.26 (± 0.87)
VAS	39	5.17 (± 1.09)	40	5.56 (± 0.81)	39	4.63 (± 1.16)
Autonomy score	42	4.48 (± 1.67)	42	5.05 (± 1.52)	42	4.64 (± 1.87)

Exposure, Calot Dissection, Cholangiography and Resection steps are rated from 1 to 4, OSATS items from 1 to 5 and VAS items from 1 to 7

*SD* standard deviation, *LCAT* Laparoscopic cholecystectomy assessment tool, *OSATS* objective structured assessment of technical skills, *VAS* Visual Analog Scale

### Differences between reviewers

Scoring results were comparable between the live reviewer and reviewer A. There was no statistically significant difference in mean scores between the live reviewer and reviewer A for all the items of the assessment tool. Scores of the live reviewer and reviewer B were significantly different in two items: OSATS (3.68 vs. 4.26 respectively; *p* = 0.04) and VAS (5.17 vs. 4.63, *p* = 0.03). There was no statistically significant difference in the LCAT.

Scores of reviewer A and reviewer B were significantly different for the same two items: OSATS (3.75 vs. 4.26 respectively; *p* = 0.001) and VAS (5.56 vs. 4.63 respectively; *p* = 0.004), whereas the LCAT scores were comparable between reviewers A and B.

### Inter-rater reliability

**Fig. 4:**
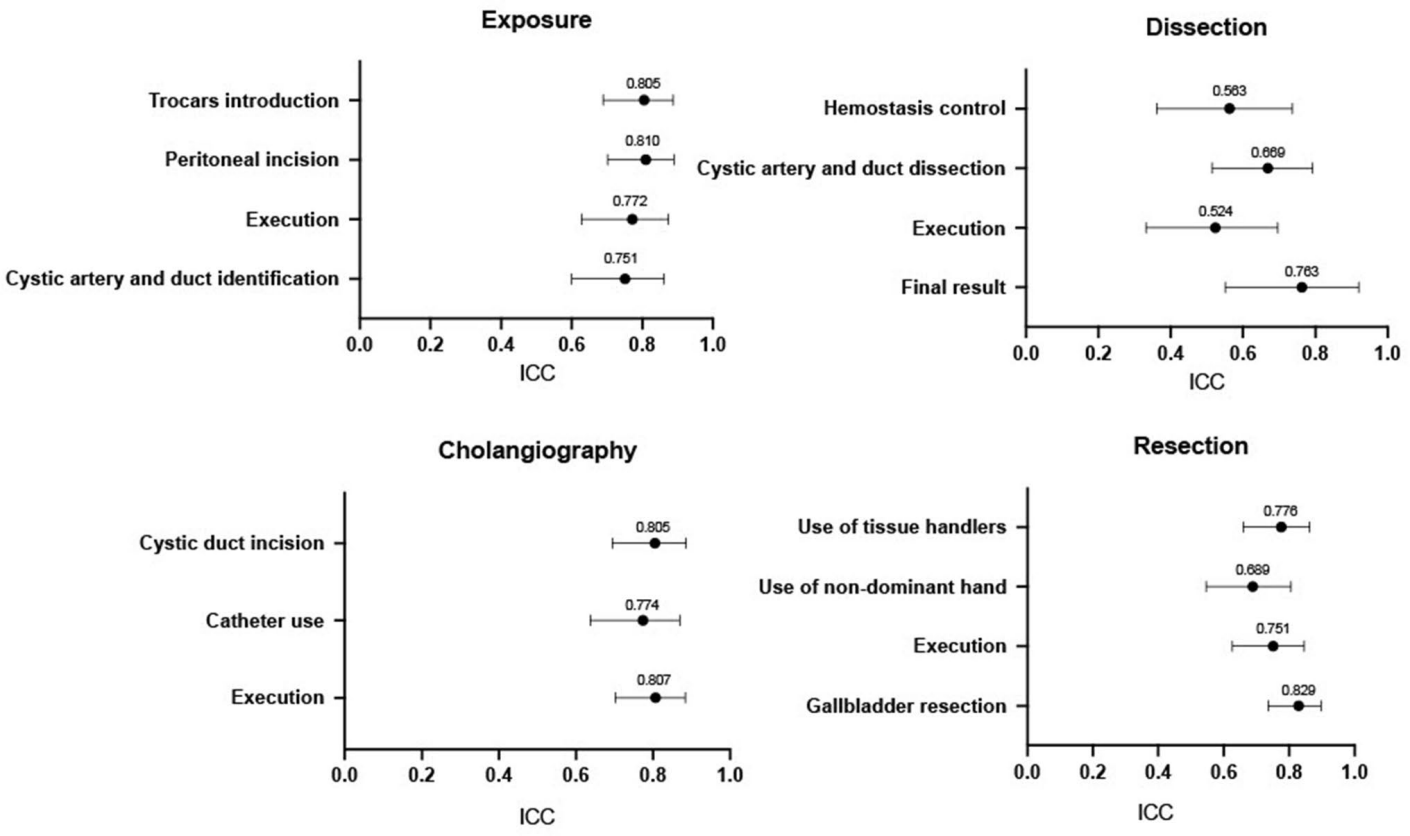
Intraclass correlation coefficients (ICC) for the four steps of the laparoscopic cholecystectomy assessment tool (LCAT). ICC value < 0.5, between 0.5 and 0.75, between 0.75 and 0.9 and > 0.90 were indicative of poor, moderate, good, and excellent reliability respectively

For the 27 scored items, the calculated inter-rater reliability coefficients (ICC) were 0.446 to 0.829 (Table 3). For one item (cholangiography interpretation), only one rating was available, so the coefficient could not be computed. 17 of the 27 coefficients showed a good reliability (0.75 to 0.9), 8 showed a moderate reliability (0.5 to 0.75) and 2 (OSATS: knowledge of instruments and OSATS: knowledge of specific procedure) scored a low correlation coefficient (0.45, 95% CI 0.361–0.615 and 0.446, 95% CI 0.248–0.633). Figure 4 shows the ICC for the cholecystectomy-specific part (LCAT) of the assessment tool.

**Table 3:** Intra-class correlation coefficient for inter-rater reliability between live reviewer and the two video recorded reviewers

	*N*	ICC	95% CI
Exposure			
Trocars introduction	35	0.805	0.690–0.888
Peritoneal incision	38	0.810	0.702–0.890
Execution	36	0.772	0.628–0,874
Cystic artery and duct identification	36	0.751	0.6–0.862
Dissection			
Hemostasis control	42	0.563	0.362–0.737
Cystic artery and duct dissection	42	0.669	0.515–0.793
Execution	42	0.524	0.332–0.696
Final result	42	0.763	0.551–0.921
Cholangiography			
Cystic duct incision	29	0.805	0.695–0.886
Catheter use	31	0.774	0.638–0.870
Execution	30	0.807	0.702–0.884
Cholangiography	NA	–	–
Resection			
Use of tissue handlers	42	0.776	0.660–0.863
Use of non-dominant hand	41	0.689	0.546–0.805
Execution	41	0.751	0.626–0.846
Gallbladder resection	42	0.829	0.736–0.897
OSATS			
Respect for tissue	42	0.797	0.690–0.877
Time and motion	40	0.821	0.724–0.892
Instrument handling	42	0.753	0.621–0.847
Knowledge of instruments	42	0.45	0.361–0.615
Use of assistants	42	0.684	0.537–0.802
Flow of operation	40	0.683	0.538–0.801
Knowledge of specific procedure	40	0.446	0.248–0.633
VAS			
Exposition	36	0.652	0.497–0.779
Calot’s triangle dissection	36	0.744	0.617–0.842
Cholangiography	29	0.787	0.675–0.870
Resection	36	0.662	0.511–0.786
Autonomy statement	42	0.806	0.702–0.882

ICC value < 0.5, between 0.5 and 0.75, between 0.75 and 0.9 and > 0.90 were indicative of poor, moderate, good, and excellent reliability respectively

*ICC* intra-class correlation coefficient, *CI* confidence interval, *OSATS* objective structured assessment of technical skills, *VAS* Visual Analog Scale

### Correlation between operative time and score

The relationship between operative time and score for each item of the assessment tool (LCAT steps, OSATS, VAS and autonomy score) during live review was investigated using Pearson product-moment correlation coefficient. There was no significant correlation between operative time and any other score (Table 4).

**Table 4:** Person correlation coefficient between operative time and score of each step of the assessment score during live-reviewed procedures.

	Pearson correlation coefficient	*N*	*p* value
Operative time	1		
Exposure	− 0.108	41	0.5
Calot dissection	− 0.377	42	0.017
Cholangiography	− 0.07	35	0.68
Resection	− 0.128	39	0.43
OSATS	− 0.238	39	0.151
VAS	− 0.233	39	0.166
Autonomy score	− 0.256	42	0.11

## Discussion

Surgical education is subject to many constraints such as reduction of trainees’ surgical volumes because of the legal limitation of working hours. Every operation performed by a young surgeon is precious and a maximum amount of teaching should be extracted from it. Objective and reliable assessment tools allow to achieve this goal by guiding and, if necessary, correcting each aspect of the intervention. We demonstrated that the implementation of a new assessment tool for laparoscopic cholecystectomy is feasible and the tool appears to be objective.

Over the last 20 years, several tools have been developed to assess general surgical skills [6], skills specific to an intervention or a combination of both [7, 8]. The creation of the LCAT was inspired by a competency assessment tool used in the United Kingdom in the residency training program for right or left laparoscopic colectomies [4]. The assessment is completed at the end of this program to confirm that the surgeon is able to perform the procedure safely. The authors of the tool demonstrated that the higher the score, the better the patient’s outcome in terms of postoperative morbidity and the number of harvested lymph nodes. We used this validated assessment tool for a more common procedure, more suitable for the assessment of non-specialized surgeons.

Feedback from a supervisor after an intervention can be infrequent. A national survey was performed in the residency programs in the United States with 998 participants. Concerning postoperative feedback, only a minority of residents reported that senior surgeons reinforced the educational points of a case with them (34%), gave positive feedback (41%), or discussed areas for improvement (37%) upon completion of an operation [9]. Formal written assessment ensures that feedback is given to the trainee immediately after the surgery. Immediate rating is more accurate than one completed later after the intervention [10]. In our study, all the assessment forms were filled directly after the procedure with full compliance. The form is quick and easy to complete.

Several general scores have been developed and applied to the laparoscopic cholecystectomy. The global operative assessment of laparoscopic surgery (GOALS) consists of general items rated from 1 to 5 with anchor explanations at the first, third and fifth points. GOALS was first applied for a single step of laparoscopic cholecystectomy, the dissection of the gallbladder from the hepatic bed, and then to the entire procedure [11, 12]. The OSATS is a validated score for general surgical skills that has been used to assess laparoscopic cholecystectomy [13]. Another general and specific assessment for laparoscopic cholecystectomy is the operative performance rating system (OPRS) [6]. It is used by the ABS [1] for the assessment of operative performance during laparoscopic cholecystectomy. The assessment is divided into both general and specific for the procedure items and the VAS ranging from 1 to 7 with anchor explanations for points 1, 4 and 7. A common flaw of these scores is the lack of procedure-specific items. A VAS can be misleading and open to interpretation by the rater. In our assessment tool, we divided the laparoscopic cholecystectomy into 16 steps with anchor explanations for each rating. This allows to standardize the procedure and identify specific areas for improvement. Each step can be corrected to maximize the safety of the patient. An additional element compared to other scores is the inclusion of the peroperative cholangiography. This step may not be systematically performed at all institutions but can be valuable in detecting common bile duct stones or delineating the anatomy of the bile ducts to avoid injuries. This skill should be acquired by any independent surgeon. It therefore seems essential to us to include cholangiography in any assessment tool for laparoscopic cholecystectomy. Objectivity and reliability of the LCAT have been demonstrated in our study as the scoring results were comparable between non-blinded live reviewers and two blinded video reviewers.

Autonomy seems to be the most challenging part to assess. In an American study, all fellowship program directors completed a survey about the residents entering their programs. They reported that 30% of the surgeons could not independently perform a laparoscopic cholecystectomy [14]. In our study, there was no evident consensus about the overall autonomy of trainees. This item showed the widest score range between all the reviewers. The autonomy level assessed by VAS should be used as information for the trainee rather than a threshold to be obtained for independent practice. Overall scores based on all the items and/or successive evaluation by different raters could better assess a trainee’s autonomy.

In our study, we compared live assessment scores with the scores given by two blinded video reviewers. Inter-rater reliability was moderate to good for most of the items in the assessment tool. No statistically significant differences in the LCAT part were noted between all three reviewers.

Video assessment simplifies the process as the evaluation is not limited by the rater’s availability. It also eliminates sources of bias as the rater is blinded to the trainee’s identity.

In our study, there were two steps the raters disagreed on: the OSATS and the VAS. In the OSATS, the mean score was different between the reviewers and there were two items which showed poor reliability: knowledge of the procedure and use of the instruments. Watching a video recording without intraoperative sound, reviewers could have difficulties to evaluate the flow of the procedure and the accuracy of the trainees’ requests for instruments. This could explain the discrepancy between the raters and suggests these items should be omitted during video rating. In the VAS part, the mean score was different between the reviewers but the individual items showed good inter-rater reliability. The VAS and the OSATS are different compared to the LCAT: not all grades on the scale have an anchor explanation. The VAS has a scale of 1 to 7 but explanations are available only for grades 1, 4 and 7. The choice of a score of 2 or 5 is therefore left to the rater’s subjective interpretation. The subjectivity of scoring in the OSATS and the VAS part could explain the differences in score between the raters. The LCAT has a clear explanation for each item leaving less room for interpretation.

The LCAT scoring shows consistency between live and video rating whereas the OSATS and VAS scores differed between reviewers.

Evaluation of laparoscopic cholecystectomy with the LCAT seems to be feasible, more objective and producing results consistent between live and video reviewers.

The operative assessment could improve the patient safety and outcome. Trainees who received feedback from an experienced surgeon after performing a cholecystectomy showed significant improvements in their operative time and efficiency of movement [15]. Giger et al. [16] demonstrated that intraoperative complications were more frequent for surgeons with experience of less than 100 laparoscopic cholecystectomies. These surgeons were classified as at risk because their patients had the highest likelihood of suffering from surgery-related complications (odds ratio [OR] 1.36; *p* = 0.0002). Murphy et al. [17] have shown that postoperative complications after laparoscopic cholecystectomy were more frequent in a non-teaching hospital. These results reinforce the need for control of surgical safety during and at the end of a training program. A successive assessment with the LCAT during the surgeon’s training could help identify any unsafe aspect of the procedure.

The laparoscopic cholecystectomy learning curve is not well defined. In their meta-analysis, Reitano et al. [18] found that thresholds for proficiency ranging from 13 to 200 procedures have been proposed. Despite laparoscopic cholecystectomy being a common procedure, there is no consensus on the minimal number of surgeries required for a trainee to become independent. In addition, reaching a certain number of surgeries does not guarantee high quality performance. De Siqueira et al. [19] reported that out of 30 trainees who had attained 50 procedures required for cholecystectomy certification, only 47% had an assessment score valid for independent practice. The LCAT could represent an alternative to evaluate surgical competence.

There are several limitations of this study. We could not exclude a selection bias, as the most challenging cases were performed entirely or at least partially by the senior surgeon and were excluded from the assessment process. In other scores, the level of guidance is a part of the assessment. The criterion of exclusion of not fully independent surgeries also explains the low number of interventions which is another study limitation. However, we found that an assessment score on a procedure done entirely by the trainee better reflects their surgical skills. It forces the trainee to perform the entire surgery under standardized guidance of the mentor and allows safe evaluation of steps with which the trainee may feel less comfortable. The assessment tool helps identify the steps in which there is room for improvement. Another limitation is exclusion of cholangiography assessment from analysis as cholangiography was not performed in every case. Finally, we could not compare assessment scores with patient outcomes as the study was not designed to include patients’ postoperative data. A further study comparing assessment scores and patient outcomes is planned.

## Conclusion

Surgical education faces new challenges posed by the legal hour restrictions and the resulting reduction of the caseload for specific surgeries. In order to improve surgical training, assessment tools are mandatory to help young surgeons progress with each procedure. We propose the LCAT as a new assessment tool specific for laparoscopic cholecystectomy.

Its implementation was feasible in our residency program. The LCAT seems to be suitable for either live or video review as both resulted in comparable scores in our study. With successive assessments using the same tool, a training program can track the improvement of each resident. An external expert could judge the adequacy of the procedure by analyzing a record of a resident at the end of their training. Further studies are required to assess the capacity of the LCAT to differentiate novice and expert (construct validity) and investigate the correlation between the LCAT score and patients outcomes.

## Supplementary Information

Below is the link to the electronic supplementary material.

Supplementary file1 (MP4 154298 KB)
